# Comparative genomic analysis of two-component regulatory proteins in *Pseudomonas syringae*

**DOI:** 10.1186/1471-2164-8-397

**Published:** 2007-10-31

**Authors:** José L Lavín, Kristoffer Kiil, Ohiana Resano, David W Ussery, José A Oguiza

**Affiliations:** 1 Departamento de Producción Agraria, Universidad Pública de Navarra, 31006 Pamplona, Spain; 2 Center for Biological Sequence Analysis, Biocentrum-DTU, The Technical University of Denmark, DK-2800 Lyngby, Denmark

## Abstract

**Background:**

*Pseudomonas syringae *is a widespread bacterial plant pathogen, and strains of *P. syringae *may be assigned to different pathovars based on host specificity among different plant species. The genomes of *P. syringae *pv. *syringae *(*Psy*) B728a, pv. *tomato *(*Pto*) DC3000 and pv. *phaseolicola *(*Pph*) 1448A have been recently sequenced providing a major resource for comparative genomic analysis. A mechanism commonly found in bacteria for signal transduction is the two-component system (TCS), which typically consists of a sensor histidine kinase (HK) and a response regulator (RR). *P. syringae *requires a complex array of TCS proteins to cope with diverse plant hosts, host responses, and environmental conditions.

**Results:**

Based on the genomic data, pattern searches with Hidden Markov Model (HMM) profiles have been used to identify putative HKs and RRs. The genomes of *Psy *B728a, *Pto *DC3000 and *Pph *1448A were found to contain a large number of genes encoding TCS proteins, and a core of complete TCS proteins were shared between these genomes: 30 putative TCS clusters, 11 orphan HKs, 33 orphan RRs, and 16 hybrid HKs. A close analysis of the distribution of genes encoding TCS proteins revealed important differences in TCS proteins among the three *P. syringae *pathovars.

**Conclusion:**

In this article we present a thorough analysis of the identification and distribution of TCS proteins among the sequenced genomes of *P. syringae*. We have identified differences in TCS proteins among the three *P. syringae *pathovars that may contribute to their diverse host ranges and association with plant hosts. The identification and analysis of the repertoire of TCS proteins in the genomes of *P. syringae *pathovars constitute a basis for future functional genomic studies of the signal transduction pathways in this important bacterial phytopathogen.

## Background

Bacterial signal transduction pathways sense the cellular external environment and regulate cellular functions in response to environmental signals. A mechanism commonly found in bacteria for signal transduction is the two-component system (TCS). Bacterial TCSs are common components of complex regulatory networks and cascades, often associated with global regulation as well as with regulation of virulence. TCS genes are typically located within the same operon encoding two signalling proteins: a transmembrane sensor histidine kinase (HK) and a cytoplasmic response regulator (RR), which may sometimes be carried by a single polypeptide to form the hybrid HKs [1]. The mechanism of signal transduction by TCS proteins is based on phosphotransfer reactions between histidine (H) and aspartate (D) residues in highly conserved signalling domains of the HKs and their cognate RRs. TCS proteins have a modular organization, which may give rise to highly complex structures, but the core structures and activities are maintained [2]. HKs are typically organized as homodimers with two functionally and structurally distinct domains: a highly variable N-terminal extracytoplasmic sensory domain, and a more conserved C-terminal cytoplasmic transmitter domain, also known as the dimerization/phosphoacceptor domain [2,3]. The sensor domain varies in length and amino acid sequence from one HK to another, conferring specificity for different environmental stimuli. In most HKs, the transmitter domain shows high sequence conservation, especially within a set of six recognizable motifs or boxes designated H, N, F, G1, G2, and G3. In particular, the H box contains an invariant H residue that is autophosphorylated in an ATP-dependent manner [4]. In contrast, CheA-like HKs that function in chemotaxis lack the sensor domain and differ from other HKs in their domain constitution and organization, where the H box of the transmitter domain resides at the N-terminal end of the protein [5-8]. LytS-like HKs also differ significantly in their domain architecture from other HKs [9-11]. RRs generally contain at least two functional domains: a conserved N-terminal receiver domain (REC domain) that is phosphorylated by the HK at a strictly conserved D residue, and one or more variable C-terminal output domains [12]. Modulation of the phosphorylated state of the RR controls either expression of the target genes or cellular behaviour. The principal type of bacterial RRs are transcription factors that regulate gene-expression with DNA-binding helix-turn-helix (HTH) output domains [1,3,12,13]. Hybrid HKs contain both a HK transmitter domain and a REC domain in a single large polypeptide, and are characterized by multi-step phosphotransfer reactions [1,7,14].

The availability of complete genome sequences for a continually growing number of bacteria has allowed the definitive assessment that TCS proteins are present in almost all bacterial species [1,8,12]. Genomic analyses demonstrate the enormous impact of TCSs on environmental adaptation of bacteria, and reveal a wide variation of HK and RR numbers between different bacterial species [7,8,12,15-20].

The bacterial plant pathogen *Pseudomonas syringae *causes disease on a variety of plant species, and strains of *P. syringae *have been classified into different pathovars depending on their host range among different plant species [21]. Infection of host plants by *P. syringae *involves growth on leaf surfaces as an epiphyte, that enters plant leaves through stomata, multiplies to large populations in the apoplast and produces disease symptoms [21,22]. *P. syringae *injects effector proteins into the cytoplasm of plant cells by means of the Hrp type III secretion system [21]. Genome comparisons indicate that *P. syringae *is significantly different from other *Pseudomonas *species [23,24], suggesting that in the adaptation to the phytopathogenic lifestyle its genome must have undergone fundamental changes without a reduction in size. The complete genomic sequences of three economically important pathovars of this plant pathogenic bacteria have been determined: *P. syringae *pv. *tomato *(*Pto*) DC3000, pv. *syringae *(*Psy*) B728a and pv. *phaseolicola *(*Pph*) 1448A [25-27]. In these genomes, over 10 to 12 % of the genes are dedicated to regulation, which may reflect the need for rapid adaptation to the diverse environments encountered during epiphytic growth, plant colonization and pathogenesis [25-27]. Genome analyses of these *P. syringae *pathovars revealed fewer extracytoplasmic function (ECF) sigma factors (10 ECF sigma factors) than in related *Pseudomonas *with different lifestyles [24]. Recently, analysis of the *Pto *DC3000 genome sequence allowed the identification of 69 HKs [28,29] and 71 RRs, 21 of which were hybrid HKs [12]. In a different study not including CheA-like HKs, 64 HKs were identified in *Pto *DC3000, 20 of which were hybrid HKs [30]. Hence, *P. syringae *requires a complex array of TCS proteins to cope with diverse plant hosts, host responses, and environmental conditions. The availability of complete genomic sequences of three different *P. syringae *pathovars makes it possible to conduct this comparative genomic study to identify and analyse the TCS proteins of *P. syringae*.

## Results and Discussion

### Distribution of TCS proteins in *P. syringae*

The putative HKs and RRs in *Psy *B728a, *Pto *DC3000 and *Pph *1448A were identified by searching the complete genome sequences for proteins containing HK and RR domains using Pfam HMM profiles. Four CheA-like HKs in each *P. syringae *genome were identified in BLASTP searches using as template the CheA HK of *E. coli *[31] (Table 1). In addition, BLASTP searches of the HKs and RRs found in each *P. syringae *pathovar against the genomes of the other two pathovars allowed the identification of additional HKs and RRs. The genomes of *P. syringae *pathovars were found to contain large numbers of genes encoding TCS proteins: 68 HKs and 93 RRs in *Psy *B728a, 69 HKs and 95 RRs in *Pto *DC3000, and 70 HKs and 92 RRs in *Pph *1448A (Table 1; see Additional File 1 and 2). The number of genes encoding hybrid HKs (REC-HKs) was 20 in *Psy *B728a, 22 in *Pto *DC3000 and 24 in *Pph *1448A (Tables 1 and 4). The HMM search method used in this work retrieved hybrid HKs as well as RRs (Table 1). No TCS proteins were identified on any of the plasmids of *Pto *DC3000 and *Pph *1448A. In recent studies, similar numbers of TCS proteins for *Pto *DC3000 have been reported: 69 HKs [28,29] and 71 RRs, 21 of which were hybrid HKs [12]; or 64 HKs in a study not including CheA-like HKs, 20 of which where hybrid HKs [30]. Although the number of ECF sigma factors in all three *P. syringae *genomes (10 ECF sigma factors) is only about half that found in other *Pseudomonas *species [24,32], the number of TCS proteins is close to that found in other *Pseudomonas *genomes [33].

**Table 1:** Distribution of HKs and RRs found in the genomes of *P. syringae *pv. *syringae *B728a, pv. *tomato *DC3000 and pv. *phaseolicola *1448A.

**HK type/RR type**	***P. syringae *pv. *syringae *B728a**	***P. syringae *pv. tomato DC3000**	***P. syringae *pv. *phaseolicola *1448A**
**Histidine kinases**			
Type IA	22	20	21
Type IB	13	15	15
Type IC	20	21	22
Type III	2	2	2
CheA-like	4	4	4
GAF-HK	6	6	5
LytS-like	1	1	1
Total HKs	68	69	70
**Response regulators**			
Stand-alone REC	12	13	10
OmpR-like	22	20	19
NarL-like	9	12	10
NtrC-like	11	11	11
LytR-like	2	2	2
PrrA-like	1	1	1
PleD-like	5	4	5
RsbU-like	2	2	2
CheB-like	3	3	3
CheC-like	1	1	1
CheW-like	2	2	2
VieA-like	1	1	1
VieB-like	1	1	1
AmiR-like	1	--	--
REC-HK (hybrid HK)	20	22	24
Total RRs	93	95	92

**Table 4:** Hybrid HK genes in the genomes of *P. syringae *pv. *syringae *B728a, pv. *tomato *DC3000 and pv. *phaseolicola *1448A.

***P. syringae *pv. *syringae *B728a**	***P. syringae *pv. tomato DC3000**	***P. syringae *pv. *phaseolicola *1448A**	**HK type**
PSYR0492	PSPTO5030	PSPPH0483	CheA-like
PSYR1292	PSPTO1482	PSPPH1362^a^	IB
PSYR1300	PSPTO1490	PSPPH1371	IC
PSYR1307	PSPTO1497	PSPPH3877	CheA-like
PSYR1585	PSPTO3900	PSPPH1568	IB
PSYR1778	PSPTO3696	PSPPH1729	IC
PSYR1939	PSPTO2129	PSPPH1905	IB
PSYR2021	PSPTO2212	PSPPH1991^a^	IB
PSYR2113	PSPTO2326^a^	PSPPH2083^a^	IB
PSYR2445	PSPTO2712	PSPPH2601	IB
PSYR2448	PSPTO2715	PSPPH2604	IB
PSYR2450	PSPTO2717	PSPPH2606	IC
PSYR2700	PSPTO2896	PSPPH2483	IC
PSYR2940	--	--	IB
PSYR3355	PSPTO3584	PSPPH3276	IC
PSYR3532	PSPTO1870	PSPPH3473	IC
PSYR3612	PSPTO1782	PSPPH3628	IB
PSYR3698/GacS	PSPTO1691	PSPPH3719	IB
PSYR3996	PSPTO4293	PSPPH4003	IC
PSYR4408	PSPTO4868	PSPPH4451	IB
--	PSPTO0896	PSPPH4242	IB
--	PSPTO0898	PSPPH0796	IB
--	PSPTO4079	--	IB
--	--	PSPPH0770	IB
--	--	PSPPH0944	IC
--	--	PSPPH1261	IC

^a ^Genes with disrupted reading frames.

HK and RR genes were scattered over the entire chromosomes of the three *P. syringae *pathovars. Conservation of the genetic organization between HK and RR genes was analysed in the genomes of *Psy *B728a, *Pto *DC3000 and *Pph *1448A allowing the identification of gene clusters containing HKs and RRs that constitute putative TCSs (Table 2). Like in other bacterial species, many *P. syringae *HKs and RRs were encoded by clusters of adjacent genes: 37 putative clusters of complete TCS genes in *Psy *B728a, 34 in *Pto *DC3000, and 33 in *Pph *1448A (Table 2). For the remaining HK or RR genes, their partner genes could not be predicted from genetic organization and, therefore, they were considered as orphan HKs or RRs. The orphan HKs were 11 in each *P. syringae *genome, and the number of genes encoding orphan RRs was very high: 36 in *Psy *B728a, 38 in *Pto *DC3000 and 35 in *Pph *1448A (Table 3). Finally, the comparative genomic analysis allowed the identification of a core of complete TCS protein orthologues among the three *P. syringae *pathovars, that is composed by 30 putative TCS clusters (HK and RR) (Table 2), 11 orphan HKs, 33 orphan RRs (Table 3), and 16 hybrid HKs (Table 4).

**Table 2:** Putative TCS gene clusters in the genomes of *P. syringae *pv.* syringae *B728a, pv.* tomato *DC3000 and pv.  *phaseolicola *1448A.

**Histidine kinase/Response regulator**			**Protein name^a^**	**Organization^b^**	**HK type**	**RR type**
***Psy *B728a**	***Pto *DC3000**	***Pph *1448A**				

PSYR0064/0063	PSPTO0126/0127	PSPPH0070/0069	FimS/AlgR	HR	LytS-like	LytR-like
PSYR0259/0258	PSPTO0329/0328	PSPPH0247/0246	EnvZ/OmpR	RH	IA	OmpR-like
PSYR0264/0263	PSPTO0335/0334	PSPPH0253/0252	--/AlgB	RH	IA	NtrC-like
PSYR0723/0722	PSPTO0824/0823	PSPPH0737/0736	PilS/PilR	RH	IC	NtrC-like
PSYR0786/0788	PSPTO0913/0915	PSPPH0805/0807	CheA1/CheY1	RH^c^	CheA-like	Stand-alone REC
PSYR0832/0831	PSPTO0965/0964	PSPPH0858/0857	--/--	HR	IC	NtrC-like
PSYR1100/1099	--/--	PSPPH1168/1167	--/--	RH^d^	IB	PleD-like
PSYR1112/1111	PSPTO1291/1290	PSPPH1180/1179	--/GltR	RH	IA	OmpR-like
PSYR1126/1127	PSPTO1306/1307	PSPPH1194/1195	--/--	RH	IA	OmpR-like
PSYR1498/1497	--/--	--/--	CopS/CopR	RH	IA	OmpR-like
PSYR1941/1940	PSPTO2131/2130	PSPPH1907/1906	--/--	HR	III	NarL-like
PSYR2031/2032	PSPTO2222/2223	PSPPH2003/2004	RhpS/RhpR	RH	IA	OmpR-like
PSYR2050/2051	PSPTO2245/2246	PSPPH2021/2022	KdpD/KdpE	HR	IA	OmpR-like
PSYR2374/2375	PSPTO2642/2643	PSPPH2510/--^e^	--/--	RH	IA	OmpR-like
PSYR2385/2384	PSPTO2652/2651	--/--	BphP2/--	HR	GAF-HK	Stand-alone REC
PSYR2867/2868	PSPTO2983/--^e^	PSPPH2377/2376	BaeS2/BaeS1	HR	IA	OmpR-like
PSYR3085/3084	--/--	PSPPH2980/--^e^	--/--	RH	IA	OmpR-like
PSYR3128/3127	PSPTO3298/3297	PSPPH3041/3040	--/--	RH	IA	OmpR-like
PSYR3211/3212	PSPTO3380/3381	PSPPH3126/3127	--/--	RH	IA	OmpR-like
PSYR3375/3374	PSPTO3604/3603	PSPPH3295/3294	--/--	RH	IA	OmpR-like
PSYR3434/3436	PSPTO1982/1980	PSPPH3360/3362	CheA2/CheY2	RH^c^	CheA-like	Stand-alone REC
PSYR3460/3459	PSPTO1955/1956	PSPPH3386/3385	FleS/FleR	HR	IC	NtrC-like
PSYR3512/3511	PSPTO1893/1894	PSPPH3454/3453	QseC/QseB	RH	IA	OmpR-like
PSYR3708/3709	PSPTO1680/1679	PSPPH3729/3730	PhoQ/PhoP	RH	IA	OmpR-like
PSYR3715/3716	PSPTO1673/1672	PSPPH3736/3737	--/RstA	RH	IA	OmpR-like
PSYR3792/3793	--/--	PSPPH1461/1460	--/CpxR	RH	IA	OmpR-like
PSYR3912/3913	PSPTO4175/4176	PSPPH3906/3907	--/--	HR	IC	NtrC-like
PSYR3964/3965	PSPTO4230/4231	PSPPH3961/3962	TctE/TctD	RH	IA	OmpR-like
PSYR3994/3995	PSPTO4291/4292	PSPPH4001/4002	--/--	HR	IC	NtrC-like
PSYR4069/4070	PSPTO4373/4374	PSPPH4074/4075	ColS/ColR	RH	IA	OmpR-like
PSYR4231/4230	PSPTO4554/4553	PSPPH4256/4255	--/--	HR	IC	PrrA-like
PSYR4619/4618	PSPTO0559/0560	PSPPH0641/0642	--/--	HR	III	NarL-like
PSYR4799/4800	PSPTO0379/0378	PSPPH4827/4828	--/--	RH	IA	OmpR-like
PSYR4821/4822	PSPTO0353/0352	PSPPH4852/4853	NtrB/NtrC	HR	IC	NtrC-like
PSYR4937/4938	PSPTO5398/5399	PSPPH0147/0146	--/--	HR	IC	NtrC-like
PSYR5033/5032	PSPTO5478/5477	PSPPH5115/5114	PhoR/PhoB	RH	IA	OmpR-like
PSYR5089/5088	PSPTO5549/5548	PSPPH5172/5171	--/--	HR	IC	LytR-like
--/--	PSPTO0785/0786	--/--	--/--	HR	IA	OmpR-like
--/--	PSPTO4705/4704	--/--	CorS/CorR	RH	IB	NarL-like
--/--	PSPTO5573/5574^e^	--/--	--/--	HR	IC	OmpR-like

^a ^Whenever a HK or RR of *P. syringae *has been assigned a function in the literature and/or an annotation in databases, the corresponding protein name is mentioned; ^b^organization of each TCS on *P. syringae *genomes (HR, 5' histidine kinase-3' response regulator; RH, 5' response regulator-3' histidine kinase); ^c ^an additional gene is located in between the RR and HK genes; ^d ^HR in *P. syringae *pv. *phaseolicola *1448A; ^e ^genes with disrupted reading frames.

**Table 3:** Orphan HK and RR genes in the genomes of *P. syringae *pv. *syringae *B728a, pv.* tomato *DC3000 and pv.  *phaseolicola *1448A.

***P. syringae *pv. *syringae *B728a**	***P. syringae *pv. tomato DC3000**	***P. syringae *pv. *phaseolicola *1448A**	**HK/RR type**
**Orphan HKs**			
PSYR1918	PSPTO2123	PSPPH1874	GAF-HK
PSYR2978	PSPTO3111	PSPPH2262	IC
PSYR3060	PSPTO3195	PSPPH2185	IC
PSYR3504/BphP1	PSPTO1902	PSPPH3446	GAF-HK
PSYR3591	PSPTO1803	PSPPH3550	IA
PSYR3773	PSPTO1606	PSPPH1480	GAF-HK
PSYR3774	PSPTO1605	PSPPH1479	GAF-HK
PSYR4089	PSPTO4395	PSPPH4095	IC
PSYR4339	PSPTO4796	PSPPH4381	IB
PSYR4373	PSPTO4833	PSPPH4416	IC
PSYR4439	PSPTO4896	PSPPH4481	GAF-HK
**Orphan RRs**			
PSYR0089	PSPTO0303	PSPPH0094	Stand-alone REC
PSYR0488/PilG	PSPTO5034	PSPPH0479	Stand-alone REC
PSYR0489/PilH	PSPTO5033	PSPPH0480	Stand-alone REC
PSYR0509	PSPTO5014	PSPPH0499	PleD-like
PSYR0781/CheB1	PSPTO0908	PSPPH0800	CheB-like
PSYR0886	PSPTO1039	PSPPH0923	CheC-like
PSYR1098	PSPTO1278	PSPPH1166	PleD-like
PSYR1139	PSPTO1323	PSPPH1207	CheW-like
PSYR1190/HrpR	PSPTO1379	PSPPH1270	NtrC-like
PSYR1191/HrpS	PSPTO1380	PSPPH1271	NtrC-like
PSYR1293	PSPTO1483	PSPPH1363	VieA-like
PSYR1294	PSPTO1484	PSPPH1364	NarL-like
PSYR1308/CheB2	PSPTO1498	PSPPH3876	CheB-like
PSYR1309/WspR	PSPTO1499	PSPPH3875	PleD-like
PSYR1384	PSPTO4027	PSPPH3800	NarL-like
PSYR1912	PSPTO2117	PSPPH1867	RsbU-like
PSYR1938	PSPTO2128	PSPPH1904	Stand-alone REC
PSYR2114	--	--	NarL-like
PSYR2115	PSPTO2330	--	Stand-alone REC
PSYR2449	PSPTO2716	PSPPH2605	Stand-alone REC
PSYR2897/GacA	PSPTO3024	PSPPH2328	NarL-like
PSYR2939	--	--	AmiR-like
PSYR3091	PSPTO3245	PSPPH2995	OmpR-like
PSYR3299	PSPTO3526	PSPPH3220	NarL-like
PSYR3433/CheB3	PSPTO1983	PSPPH3359	CheB-like
PSYR3451	PSPTO1964	PSPPH3377	RsbU-like
PSYR3461/FleQ	PSPTO1954	PSPPH3387	NtrC-like
PSYR3486	PSPTO1927	PSPPH3413	CheW-like
PSYR3496	PSPTO1911	PSPPH3428	VieB-like
PSYR3589	PSPTO1806	PSPPH3547	OmpR-like
PSYR3890	PSPTO4151	PSPPH1374	NarL-like
PSYR4376	PSPTO4836	PSPPH4419	NarL-like
PSYR4377	PSPTO4837	PSPPH4420	PleD-like
PSYR4388	PSPTO4848	PSPPH4431	Stand-alone REC
PSYR4701	PSPTO0472	PSPPH4737	Stand-alone REC
PSYR5036	PSPTO5482	PSPPH5118	Stand-alone REC
--	PSPTO0897	PSPPH4241	NarL-like
--	PSPTO2329^a^	--	Stand-alone REC
--	PSPTO4080	--	NarL-like
--	PSPTO4706/CorP	--	NarL-like
--	--	PSPPH0778	NarL-like

^a ^Genes with disrupted reading frames.

### Classification of HKs

**Figure 1:**
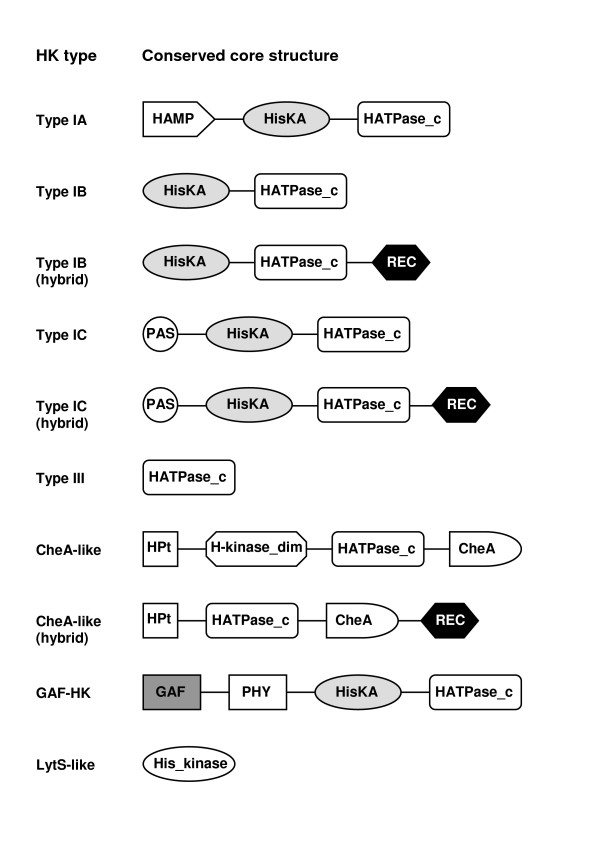
**Schematic representation of the conserved core structures found in *P. syringae *HK types**. The domains are not drawn to scale. HAMP, domain found in HKs, Adenylyl cyclases, Methyl binding proteins and Phosphatases (PF00672); HisKA, HK dimerization/phosphoaceptor domain (PF00512); HATPase_c, HK-type ATPase catalytic domain (PF02518); REC, receiver domain (PF00072); PAS, signal sensor domain (PF00989); HPt, Histidine-containing Phosphotransfer domain (PF01627); H-kinase_dim, HK homodimeric domain (PF02895); GAF, signal sensor domain (PF01590); PHY, phytochrome domain (PF00360); His_kinase, region within bacterial HKs (PF06850).

HKs have been classified on the basis of phylogenetic analyses and the sequence relationships of the residues surrounding the H-box [7,8,17,34]. Furthermore, several new domains with putative biological functions have been described in HKs, and domain architecture has proven particularly informative for analysing multi-domain proteins involved in signal transduction [2,11,12,35]. The phylogenetic analysis and examination of the region around the H box of *P. syringae *HKs showed that three of the five major HK types found in *E. coli *[8] were present in *P. syringae*: Type I (IA, IB, IC), III, and CheA-like HKs (Table 1; see Additional File 1). In contrast, Type II and IV HKs were totally absent from *P. syringae*. However, the LytS-like HK FimS/AlgZ and HKs containing GAF domains did not cluster within any of the defined HK types of *E. coli *[8], and formed two separate HK groups: LytS-like HKs and GAF-HKs. GAF sensor domains are commonly found cytoplasmic signalling domains in the N-terminal region of HKs [2,34], and appear to act as binding sites for small ligands, such as cyclic nucleotides (cAMP and cGMP) and small molecules, which modulate the catalytic activity of the target protein [36,37]. In addition, analysis of domain architecture of *P. syringae *HKs showed a conserved core structure for each HK type in *P. syringae *(Figure 1). The conserved core of Type III HKs and LytS-like HKs only had a HK-like ATPase (HATPase_c) catalytic domain and a His_kinase domain, respectively. The conserved core of CheA-like HKs contained a C-terminal CheA regulatory domain but lacked the HisKA domain. The conserved core of Type I HKs and GAF-HKs had a central region with HisKA and HATPase_c domains fused to additional domains on the N-terminal end: a HAMP domain in Type IA, a PAS domain in Type IC, and GAF plus phytochrome (PHY) binding domains in GAF-HKs (Figure 1).

Orphan HKs fell into two HK types: Type I (IA, IB and IC), and GAF-HKs (Table 3); and hybrid HKs of *P. syringae *belong either to the Type I (IB and IC) or CheA-like HKs (Table 4). PSYR3504 (BphP1) and PSYR2385 (BphP2) HKs have been previously described as bacteriophytochromes (BphPs) that belong to the HWE_HK family [4,38]. Similar to other BphPs, the *bphP1 *(PSYR3504) gene of *P. syringae *pathovars is located in an operon downstream from a *bphO *gene, encoding a putative heme oxigenase.

### Classification of RRs

RRs show a great variety of output domains and domain combinations. Recently, bacterial and archaeal RRs have been classified into families based in their domain architectures [12]. RRs typically consist of an N-terminal REC domain fused to a C-terminal HTH DNA-binding output domain (OmpR, NarL, NtrC, LytR, AraC, Spo0A, Fis, YcbB, RpoE, and MerR) that activates or represses transcription of specific target genes [2,12]. In addition, prokaryotic genomes encode a variety of RRs with unusual domain organization: RRs with enzymatic output domains (GGDEF, EAL, HD-GYP, CheB, CheC, PP2C, and HisKA), RRs with RNA-binding output domains (ANTAR and CsrA), RRs with protein- or ligand-binding output domains (CheW, PAS, GAF, TPR, CAP_ED, and Hpt), RRs with the REC domain as a stand-alone module, and RRs with domains of unknown function [12]. The RRs identified from the genomes of *P. syringae *pathovars were assigned to these different RR families [12] according to the domain architecture and phylogenetic analysis (Table 1; see Additional File 2).

Bacterial RRs without a REC domain are extremely rare, but a number of enhancer-binding proteins (EBPs) lack the REC domain and normally function as RRs [39]. EBPs are involved in the activation of the bacterial transcription by interaction with the sigma-54 RNA polymerase holoenzyme [40]. In *P. syringae*, the HrpR and HrpS proteins show a high sequence similarity to the NtrC family of transcriptional RRs and have been previously identified as unusual EBPs lacking the N-terminal REC domain; however, similar to other EBPs, they retain the domain that interacts with the sigma-54 RNA polymerase holoenzyme plus the C-terminal DNA-binding domain [39-42]. In addition, the NarL-like RR CorP of *Pto *DC3000 that is involved in the regulation of coronatine biosynthesis [43,44] also lacks the REC domain. Thus, HrpR, HrpS and CorP proteins were not identified during the search of RRs in *P. syringae *genomes with the HMM profile that targets the RR REC domain, nevertheless these proteins were considered orphan RRs (Table 3).

### Differences in TCS genes among pathovars that may contribute to plant host specificity

A close analysis of the distribution of genes encoding TCS proteins revealed that there are important differences in TCS proteins among the three pathovars of *P. syringae *that may contribute to their diverse host ranges and association with particular host plants. A number of the identified TCS genes were unique to each *P. syringae *pathovar without counterparts in the other two pathovars. The *corRSP *regulatory region (PSPTO4704-4706) of coronatine biosynthesis and the *copRS *TCS (PSYR1497/1498) regulating copper resistance were only present in *Pto *DC3000 and *Psy *B728a, respectively. Other TCS genes unique to each *P. syringae *pathovar were: PSYR2114, PSYR2939, PSYR2940 and PSYR3084 in *Psy *B728a; PSPTO0785/0786, PSPTO2329, PSPTO4079, PSPTO4080 and PSPTO5573/5574 in *Pto *DC3000; PSPPH0770, PSPPH0778, PSPPH0944 and PSPPH1261 in *Pph *1448A. The unique hybrid HKs PSPPH0770 and PSPPH0944 were flanked by transposases. However, the unique RRs PSPTO2329 and PSPTO5574 were disrupted by transposases [25,27], and it is unlikely that these genes encode functional products. Finally, 11 TCS proteins were only shared between two of these *P. syringae *pathovars.

Variations among *P. syringae *pathovars were also produced by the insertion of mobile genetic elements or point mutations in TCS genes resulting in disrupted reading frames. PSPTO2326 and PSPPH2083 encoded truncated hybrid HKs by comparison with the length of their orthologue PSYR2113 (Table 4) that is located next to the unique RR PSYR2114. PSPTO2326 and PSPPH2083 were located adjacent to a transposase and to a site-specific recombinase, respectively. Probably these elements caused the disrupted hybrid HKs and the lack of PSYR2114 orthologues in *Pto *DC3000 and *Pph *1448A. Similarly, PSPTO2983 (*baeS2*) and PSPPH2510 encoded truncated HKs compared to the length of their *P. syringae *orthologues, and PSPPH2980 was interrupted by an ISPsy18 transposase. PSPTO2983, PSPPH2510 and PSPPH2980 HKs were unpaired without a RR gene in its vicinity, whereas their *P. syringae *orthologues are located on TCS gene clusters with adjacent RRs (Table 2).

Although the PSPPH1362 gene was disrupted by an authentic frameshift, *Psy *B728a (PSYR1292) and *Pto *DC3000 (PSPTO1482) orthologues encoded intact hybrid HKs with similarity to BvgS of *Bordetella *species that controls the regulation of many virulence factors [45]. In each pathovar, these hybrid HK genes were adjacent to orphan RR genes transcribed in the same direction (PSYR1293, PSPTO1482 and PSPPH1363), and their encoded proteins exhibited significant homology to the PvrR RR of *P. aeruginosa *PA14 which controls antibiotic susceptibility and biofilm formation [46], and to the virulence related protein VieA of *Vibrio cholerae *[47].

## Conclusion

In this article we present a thorough analysis of the identification and distribution of TCS proteins among the sequenced genomes of *P. syringae*. A large set of TCS proteins is required for the capacity of *P. syringae *to detect and adapt to changing environments during plant association and pathogenesis. Moreover, *P. syringae *has been isolated from non-plant environments such as river epilithon (rock-attached biofilms) [48] in which TCS proteins may have also important regulatory roles. *P. syringae *pathovars posses between 68–70 HKs and 92–95 RRs (Table 1), however there is little information describing their regulatory functions and the major part of these TCS proteins is uncharacterized. Many of the TCS proteins investigated so far in *P. syringae *have been shown to be involved in plant pathogenicity and association with host plants. The orphan RRs HrpR and HrpS are involved in a complex regulatory cascade that activates the transcription of the Hrp type III secretion genes and all known effector genes [42,49]. Expression of the type III secretion genes and effector genes is also regulated by the particular TCS GacA/GacS [50] and the RhpRS system [51]. Furthermore, the GacA/GacS system controls the expression of a variety of virulence factors, including protease and syringomycin biosynthesis [52]. The TCS CopRS and the modified CorRSP system regulate resistance to copper [53] and coronatine synthesis [43,44], respectively. Finally, the hybrid HK PSPTO2896 contains an N-terminal LOV (light, oxygen, or voltage) domain and is blue-light-activated [54].

Bacteria with large genomes are disproportionately enriched in regulatory proteins involved in transcription control and signal transduction compared to medium and small-size genomes, and typically have complex regulatory networks relative to bacteria with smaller genomes [55-57]. The existence of large numbers of HKs and RRs in *P. syringae *strongly suggests that TCS proteins play important regulatory roles in the adaptation of this bacterium to different plant and non-plant environments. Comparative genomics of closely related species of pathogenic bacteria represents a powerful tool for the identification of genes potentially involved in host specificity and pathogenesis. The availability of the genome sequences of *Pto *DC3000, *Psy *B728a and *Pph *1448A provides us with the unique capability of comparing the complement of TCS proteins in these *P. syringae *pathovars that differ in host range and other interactions with plants. This comparative genomic analysis reveals a core of orthologues and important differences in TCS genes between *P. syringae *pathovars. It is especially worth noting the high number of genes encoding orphan HKs and RRs in these genomes. Moreover, differences in the repertoires of TCS proteins are likely to facilitate the adaptation of *P. syringae *pathovars to different plant hosts and/or could be responsible for the different disease characteristics induced. Consequently, the TCS proteins unique to each *P. syringae *pathovar are interesting targets for future investigations to identify TCS proteins involved in the different host ranges and/or plant pathogenesis. However, the challenge remains to associate these differences in TCS proteins to specific traits of *P. syringae *pathovars. Additionally, pathovar-specific differences in gene content might be used to design targeted approaches for disease control and could allow the precise PCR-based diagnosis of bacterial diseases [58].

Analysis of the regulatory functions, molecular mechanisms and signal transduction pathways of TCS proteins should contribute to the understanding of the complex events that occur in *P. syringae *during pathogenesis and adaptation to different plant hosts and different non-plant environments. Rapid progress in the study of TCS proteins is being made by the combination of molecular genetic approaches with genome-scale analysis [59]. Genetic and biochemical studies are necessary to further explore the signal transduction pathways mediated by some of these TCS proteins at the molecular level: construction and analysis of deletion mutants in TCS genes in order to determine the signals sensed by the HK and the targets for the RR of each system. In addition, the application of more extensive analysis with global methods, such as DNA microarray studies reported for *B. subtilis *[60] and *S. pneumoniae *[61], might allow defining the regulons and the potential regulatory functions of TCS proteins in response to environmental signals. Furthermore, unravelling these signal transduction pathways could potentially lead to the design of innovative strategies to control *P. syringae*. In conclusion, this comparative genomic analysis constitutes a basis for future functional genomic analysis of *P. syringae *to establish which TCS proteins participate in the pathogenesis and the adaptation to different plant and non-plant environments.

## Methods

### Identification of TCS proteins in *P. syringae *genomes

The identification of HKs and RRs is based on the computational domain analysis of protein sequences. The approach used to identify putative HKs and RRs from the complete genome sequences of *Psy *B728a, *Pto *DC3000 and *Pph *1448A was similar to that described previously [33] with slight modification. Briefly, five different HMM profiles (accession numbers PF00512, PF07568, PF07730, PF07536 and PF06580) were found in Pfam database that target different families of HKs (HisKA, HisKA_2, HisKA_3, HWE_HK and His_kinase). The HWE_HK domain is defined by the absence of a recognizable F box, and the presence of a highly conserved H residue and a WxE motif within the N and G1 boxes of the C-terminal transmitter domain, respectively [4]. These five different HMM profiles were used to recognize the different HKs in the *P. syringae *genomes, and hits with a E-value below a selected cut-off (10^-6^) were extracted. A profile HMM downloaded from Pfam protein families database [62], which targets the RR REC domain (accession number PF00072), was used to recognize the RRs in each *P. syringae *genome. Hits with an E-value below a selected cut-off (10^-12^) were extracted. Additionally, the CheA HK of *Escherichia coli *[31] was used as template in BLASTP searches to identify CheA-like HKs in the *P. syringae *genomes and hits with an E-value below a selected cut-off (10^-10^) were extracted. Hybrid HKs (REC-HKs) were determined by the presence of complete HK transmitter and REC domains in a single protein. Detection of orthologues of the identified HKs and RRs between the genomes of *Psy *B728a, *Pto *DC3000 and *Pph *1448A was determined by BLASTP [63] based on the reciprocal best hits of each *P. syringae *genome against each other genome, completed by the phylogenetic analyses. Finally, functional domains of the HKs and RRs were identified by search the Conserved Domain Databases (CDD) with Reverse Specific Position BLAST [64].

### Sequence alignment and phylogenetic analysis

Multiple sequence alignments and phylogenetic trees of HKs and RRs were constructed using the ClustalW program [65], and aligned sequences were imported into the MEGA 3.1 program [66] where phylogenetic trees were inferred. Default parameters were used. Phylogenetic trees were subdivided into groups of orthologues, and co-clustering with members of specific TCS proteins allowed a definitive assignation to a given HK type or RR family.

## List of abbreviations

TCS: two-component system

HK: histidine kinase

RR: response regulator

HMM: Hidden Markov Model

HTH: helix-turn-helix

ECF: extracytoplasmic function

EBP: enhancer-binding protein

*Psy*: *Pseudomonas syringae *pv. *syringae*

*Pto*: *P. syringae *pv. *tomato*

*Pph*: *P. syringae *pv. *phaseolicola*

REC: receiver

PHY: phytochrome

LOV: light, oxygen, and voltage

## Authors' contributions

DWU and JAO designed and coordinated the project. JLL, KK and OR performed the bioinformatics studies and interpreted the results. JAO wrote the manuscript. All authors have read and approved the final manuscript.

## Supplementary Material

Additional file 1 HKs in the genomes of *P. syringae *pv. *syringae *B728a, pv. *tomato *DC3000 and pv. *phaseolicola *1448A. Click here for file

Additional file 2 RRs in the genomes of *P. syringae *pv. *syringae *B728a, pv. *tomato *DC3000 and pv. *phaseolicola *1448A. Click here for file
